# Editorial: Breeding Innovations in Underutilized Temperate Fruit Trees

**DOI:** 10.3389/fpls.2021.799233

**Published:** 2021-11-24

**Authors:** Giuseppe Ferrara, Agata Gadaleta, Malli Aradhya, J. Iñaki Hormaza, Maria Luisa Badenes

**Affiliations:** ^1^Department of Plant, Soil and Food Science, University of Bari ‘Aldo Moro’, Bari, Italy; ^2^Department of Environmental and Territorial Sciences, University of Bari ‘Aldo Moro’, Bari, Italy; ^3^Agricultural Research Service, United States Department of Agriculture, Washington, DC, United States; ^4^Subtropical Fruit Crops Department, IHSM La Mayora – CSIC – UMA, Málaga, Spain; ^5^Department of Citriculture and Crop Production, Instituto Valenciano de Investigaciones Agrarias, Moncada, Spain

**Keywords:** fig (*Ficus carica* L.), pomegranate (*Punica granatum* L.), walnut (*Juglans regia L*.), carob (*Ceratonia siliqua* L.), pear, feijoa (*Acca sellowiana Berg*.), *Citrinae*

The recent growing interest in minor species (i.e., fig, pomegranate, feijoa, etc.) has recently driven new research on breeding and genetics to address producer and consumer traits. Since these species have received little attention from the scientific community, they were less improved *via* conventional breeding, and lacked detailed genomic information on important traits. This lack of data, together with a general poor genetic knowledge of these species, has limited a wider cultivation of varieties with improved characteristics.

For these reasons, and with the objective to increase the interest of scientists, farmers, and consumers for these fruits, this Research Topic “*Breeding Innovations in Underutilized Temperate Fruit Trees*” comprises biochemical, morphological, and genetic studies on some minor species regarding fruit trait variation, resistance, evolution, or sex determination.

In this context, Marcotuli et al. examined the mechanisms behind the bud evolution toward breba or main crop in fig (*Ficus carica* L.), since this aspect remains unclear. The X-ray images of buds showed a great structural similarity between breba and the main crop during the initial stages of development, but breba inflorescence differentiation was completed in two seasons whereas that of main crop started at the end of winter and was completed within 2–3 months in the same season. The higher expression of floral homeotic protein AGAMOUS in breba compared to the main crop may indicate a role of these fruits on staminate flowers' production for pollination of the main crop, as profichi act in the caprifig. Within the same species (*Ficus carica* L.) and for sexual determination, Ikegami et al. analyzed the *FcRAN1* gene (during a breeding program for the selection of female plants) strongly associated with the sex phenotype. A male-biased segregation ratio distortion was obtained in 12 F1 populations, suggesting some genetic factor(s) affecting it. A comparison between the annotated genes and the genes required for normal embryo or gametophyte development and function identified several candidate genes responsible for the segregation distortion in fig. Following the same topic, Wang et al. hypothesized an early sex-identification method to improve breeding efficiency. The use of a deletion as a forward primer, a newly established AG-Marker, was as accurate as the RAN1-Marker, and provided the identification of male plants, giving new clues to understanding *Ficus* sex determination. Moving toward another attracting species, i.e., pomegranate (*Punica granatum* L.), Trainin et al. investigated the black peel color of some pomegranate varieties. Biochemical analysis revealed that delphinidin is highly abundant in the peel of black varieties and the pattern of anthocyanin accumulation is different from that of other pomegranates with red or pink colors of the peel. Genetic analysis of an F2 population segregating for the black phenotype revealed that it is determined by a single recessive gene. Pomegranate was also studied by Goudappa Patil et al. with regards to the SSR of “Tunisia” pomegranate variety. There was a positive trend in chromosome length and the SSR abundance, as marker density, enhanced with a shorter chromosome length. Examination of the distribution of SSR motif types within a chromosome suggested the abundance of hexanucleotide repeats in each chromosome followed by dinucleotides. A comprehensive set of highly polymorphic genome-wide SSRs was successively developed and tested. These chromosome-specific SSRs could serve as a powerful genomic tool to leverage future genetic studies, germplasm management, and genomics-assisted breeding in pomegranate varieties. Some evolutionary aspects of pear were investigated by Kumar et al. who put a light on runs of homozygosity (ROH) in self-incompatible plants, in particular Asian pears, European pears, and interspecific hybrids using genotyping-by-sequencing. The observed ROH patterns suggested that systematic breeding of European pears would have started earlier than Asian pears. Fruit trait variation in Persian walnut (*Juglans regia* L.) was addressed by Bernard et al. who conducted a genome-wide association study (GWAS) using multi-locus models in a panel of 170 accessions of *J. regia* to elucidate the genetic determinants of fruit quality traits in walnut toward the breeding of new varieties. The authors proposed several candidate genes involved in nut characteristics, such as a gene coding for a beta-galactosidase linked to several size-related traits and known to also be involved in fruit development in other species. With regards of fruit traits, Kyratzis et al. investigated the germplasm of an ancient species, the carob (*Ceratonia siliqua* L.), on the island of Cyprus. The domestic germplasm varies both in terms of pod morphology and composition, reflecting the genetic and physiological characteristics of both grafted and non-grafted accessions, and possibly the impact of agro-environmental conditions. Morphological traits, such as seeds-to-pod weight ratio, pod width, and thickness, were principally under genetic control. Contrarily, chemical compounds, particularly total phenolic content, including condensed tannins, *in vitro* antioxidant capacity, and to a lesser extent gallic acid, organic acids, sugars (glucose and fructose), and minerals were more under agro-environmental control. In the Southern Hemisphere, Quezada et al. worked on feijoa (*Acca sellowiana* Berg.), a fruit tree species native to Uruguay and Brazil. A high-density composite genetic linkage map of feijoa was constructed using two genetically populations. Genotyping by sequencing (GBS) approach was successfully applied for developing single nucleotide polymorphism (SNP) markers. They used both the reference genome of the closely related species *Eucalyptus grandis* and a *de novo* pipeline to construct a composite map. A novel approach for the construction of composite maps where the meiosis information of individuals of two connected populations is captured in a single estimator is described. The topic of resistance was carried out by Alves et al. in order to find sources of genetic resistance to Huanglongbing (HLB)-associated “*Candidatus Liberibacter asiaticus*” (Las), one of the most destructive diseases of citrus. Some genotypes from subtribe *Citrinae*, sexually incompatible but graft-compatible with Citrus, may provide new rootstocks able to restrict bacterial titer in the canopy. Authors tested for Las resistance a wide collection of graft-compatible *Citrinae* species using an aggressive and consistent challenge-inoculation and evaluation procedure. *Eremocitrus glauca* and Papua/New Guinea Microcitrus species as well as their hybrids resulted in full resistance, opening the way for using these underutilized genotypes as Las resistance sources in breeding programs.

## Author Contributions

All authors listed have made a substantial, direct, and intellectual contribution to the work and approved it for publication.

## Conflict of Interest

The authors declare that the research was conducted in the absence of any commercial or financial relationships that could be construed as a potential conflict of interest.

## Publisher's Note

All claims expressed in this article are solely those of the authors and do not necessarily represent those of their affiliated organizations, or those of the publisher, the editors and the reviewers. Any product that may be evaluated in this article, or claim that may be made by its manufacturer, is not guaranteed or endorsed by the publisher.

